# *BmPAH* Catalyzes the Initial Melanin Biosynthetic Step in *Bombyx mori*

**DOI:** 10.1371/journal.pone.0071984

**Published:** 2013-08-26

**Authors:** Ping Chen, Li Li, Jiying Wang, Haiyin Li, Yan Li, Yin Lv, Cheng Lu

**Affiliations:** 1 State Key Laboratory of Silkworm Genome Biology and College of Biotechnology, Southwest University, Chongqing, China; 2 College of Biotechnology, Southwest University, Chongqing, China; U. Kentucky, United States of America

## Abstract

Pigmentation during insect development is a primal adaptive requirement. In the silkworm, melanin is the primary component of larval pigments. The rate limiting substrate in melanin synthesis is tyrosine, which is converted from phenylalanine by the rate-limiting enzyme phenylalanine hydroxylase (PAH). While the role of tyrosine, derived from phenylalanine, in the synthesis of fiber proteins has long been known, the role of PAH in melanin synthesis is still unknown in silkworm. To define the importance of PAH, we cloned the cDNA sequence of *BmPAH* and expressed its complete coding sequence using the Bac-to-Bac baculovirus expression system. Purified recombinant protein had high PAH activity, some tryptophan hydroxylase activity, but no tyrosine hydroxylase activity, which are typical properties of PAH in invertebrates. Because melanin synthesis is most robust during the embryonic stage and larval integument recoloring stage, we injected *BmPAH* dsRNA into silkworm eggs and observed that decreasing *BmPAH* mRNA reduced neonatal larval tyrosine and caused insect coloration to fail. *In vitro* cultures and injection of 4^th^ instar larval integuments with PAH inhibitor revealed that PAH activity was essential for larval marking coloration. These data show that *BmPAH* is necessary for melanin synthesis and we propose that conversion of phenylalanine to tyrosine by PAH is the first step in the melanin biosynthetic pathway in the silkworm.

## Introduction

Animal and insect body color and markings serve diverse functions, including mimicry, warning, courtship, thermoregulation, and roles in various adaptive processes [1]–[2]. Melanin, a chief component of animal pigmentation found in hair, skin and eyes, is necessary for free radical absorption as it shields the host from various types of ionizing radiation including UV light [3]. Melanin is also associated with some less common human diseases such as albinism, vitiligo, and melanoma. In invertebrates, melanin is associated with the formation of body color and marking, in addition to its role in many important physiological events including cuticle hardening, wound healing, and melanization of microbes and parasites during immune responses [4]–[8].

Early research suggests that melanin biogenesis occurs by the hydroxylation of the amino acid L-tyrosine (tyrosine) to L-dihydroxyphenylalanine (dopa), which, in turn, is oxidized to dopaquinone [9]. In insects, tyrosine hydroxylase (TH, EC 1.14.16.2) catalyzes the hydroxylation of tyrosine to dopa, and in many insects, this is the first and rate-limiting step for cuticular melanization [4], [10]–[12]. Dopa is then decarboxylated to dopamine, which along with dopa, is subsequently oxidized to their corresponding quinones [11]–[13] and finally converted to 5,6-dihydroxyindole(DHI) to produce melanin [14]–[15]. In *Manduca sexta*, dopamine is the primary precursor for melanin synthesis [16]. Despite a common pathway for melanin biosynthesis between vertebrates and invertebrates, significant differences have been observed – particularly with respect to the enzymes involved in the conversions and the type of melanin synthesized [14], [17].

Tyrosine is a normal dietary component, although it can be obtained from L-phenylalanine (phenylalanine) by phenylalanine hydroxylase (PAH, EC 1.14.16.1) [18], an important member of the aromatic amino acid hydroxylase (AAAH) family [19]. In mammals, defects in PAH activity give rise to elevated plasma phenylalanine and phenylketonuria (PKU) [20]. Furthermore, PAH dysfunction in the epidermis has been implicated as a causative factor for vitiligo [21]–[23]. Therefore, conversion of phenylalanine to tyrosine by PAH is critical, and in several insects, dietary tyrosine is stored in an inert form in the hemolymph [24]–[26]. Endogenous synthesis of tyrosine is essential for melanin formation, which is responsible for several physiological events, particularly during the non-ingestion insect developmental stages such as embryonic or molting stages. Previous studies with mosquitoes suggest a role for PAH in melanin formation during the immune response [8], [27].

In lepidopterans, body color has been shown to be regulated by 20 hydroxyecdysone-induced melanin synthesis [13], [28]–[29] and the roles of several enzymes involved in color pattern formation in swallowtail butterflies have been previously reported [12], [30]. However, little is known about the involvement of PAH in melanin formation for insect body and marking coloration.

In the silkworm, *Bombyx mori*, the role of tyrosine in the synthesis of fibrous proteins is well established [31]. However, the PAH that catalyzes the conversion of phenylalanine to tyrosine has not been characterized; thus, its role in melanin synthesis in the silkworm is unclear. To characterize PAH in this lepidopteran model, we chose the embryonic and larval integument recoloring stages, when melanin synthesis is the most abundant. These non-ingestion stages are also optimal to study the mechanisms of melanin anabolism and to identify genes that encode enzymes involved in the pathway.

Here, we report the critical role of *BmPAH* in melanin synthesis based on the ability of BmPAH expressed *in vitro* to convert phenylalanine to tyrosine. We used RNAi-mediated gene silencing to investigate coloring in embryos, and cultured *in vitro* and injected with PAH inhibitor to investigate markings on larvae to elucidate the role of BmPAH in melanin formation.

## Results

### 2.1 Identification and biochemical characterization of *BmPAH*

**Figure 1:**
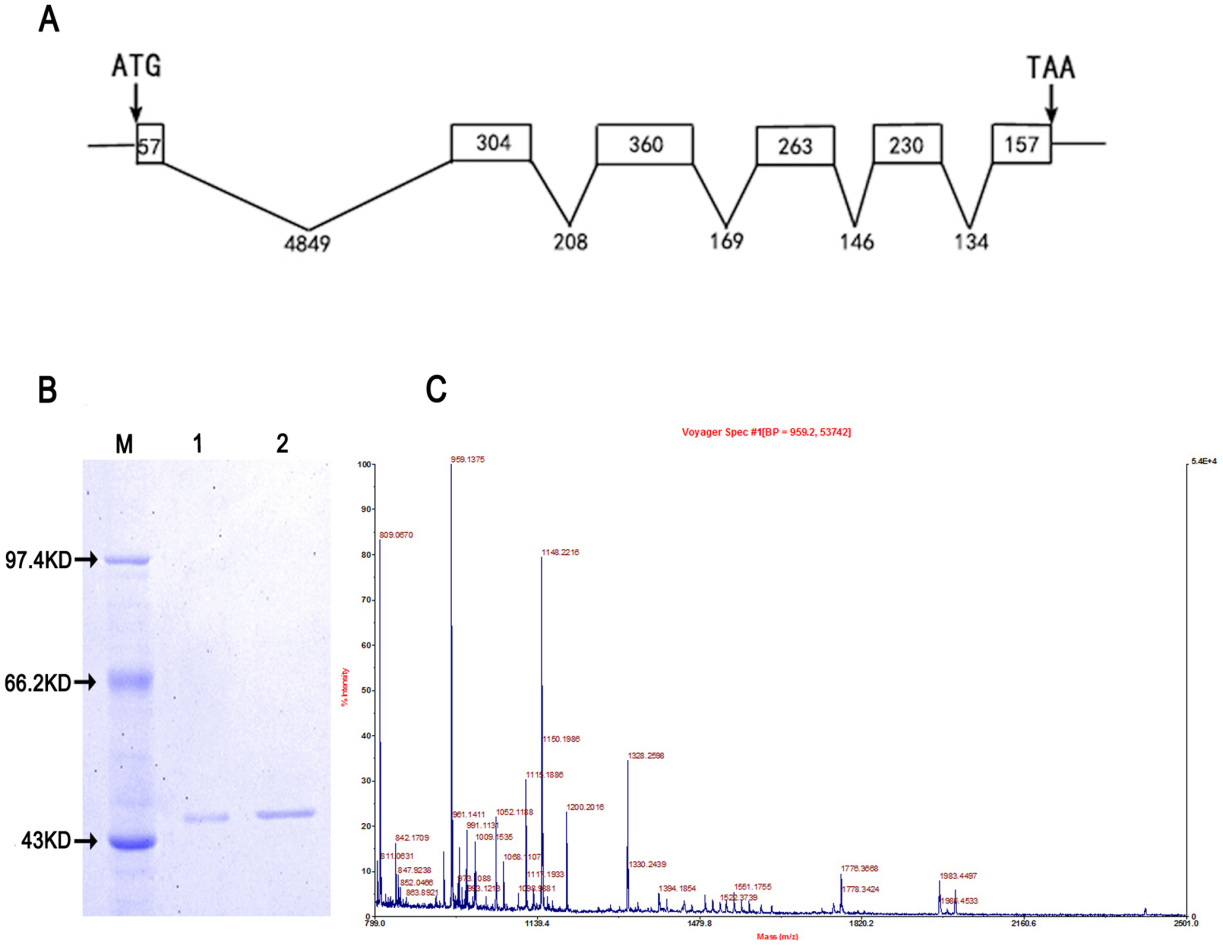
The structure of *BmPAH* gene and identification of BmPAH expressed *in vitro*. A: The structure of the *BmPAH* gene. Exons are represented by boxes, with the number indicating the length of individual exon; the length of the introns is also shown. B: 12% SDS-PAGE of proteins stained with Coomassie brilliant blue. A protein of approximately 52 kDa was recognized. lane 1: protein standard; lane 2: 0.3 µg purified protein; lane 3: 0.6 µg purified protein. C: Peptide mass fingerprint by MALDI-TOF-MS.

Conversion of phenylalanine into tyrosine is necessary to elucidate whether *BmPAH* participates in melanin biosynthesis. To this end, we isolated *BmPAH* and cloned the 1433 bp cDNA sequence of *BmPAH* containing the 1371 bp ORF (GU953670). The BmPAH gene had six exons and five introns (Figure 1A), and its predicted amino acid sequence shared 72% similarity with *Henna* from *Drosophila melanogaster* and 59% with human PAH. Without the signal peptide, it contained an N-terminal ACT regulatory domain and a C-terminal catalytic domain, which were also observed in humans and *D. melanogaster*.

Furthermore, the complete coding sequence of *BmPAH* was expressed in the Bac-to-Bac Baculovirus Expression System because protein processing and modification in this system is similar to the endosomatic environment of the silkworm. The molecular weight of the expressed recombinant protein was ∼52 KDa as estimated by electrophoresis, a finding that was consistent with the predicted weight of BmPAH (Figure 1B). Protein purity was ≥90% and the purified protein was identified by peptide mass fingerprinting (Figure 1C). The peptide fingerprint masses were analyzed by General Protein/Mass Analysis for Windows software (GPMAW), which showed coverage of matched peptides  = 31.1% and matched peptides  = 15 (average difference  = 0.38). This indicated that the protein expressed and purified *in vitro* was the product encoded by the BmPAH gene.

**Figure 2:**
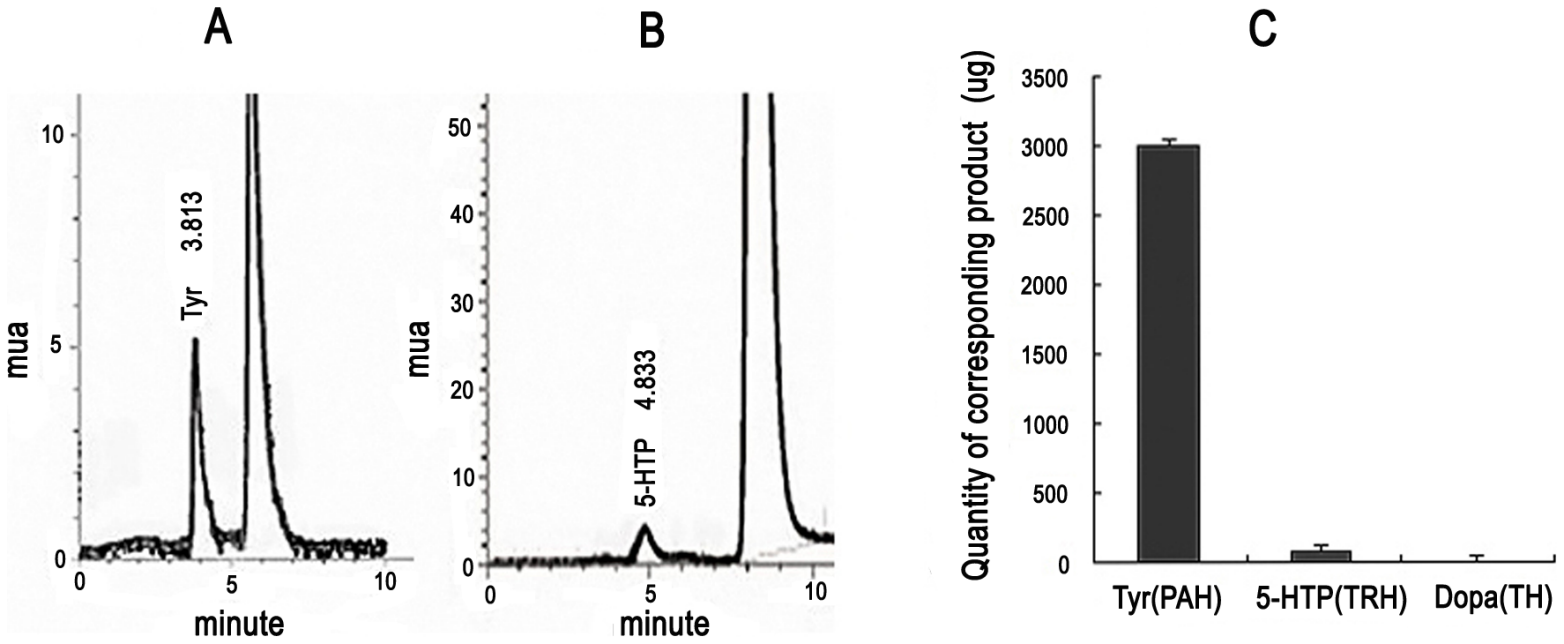
Activity analysis of BmPAH protein expressed *in vitro.* Tyr: tyrosine; 5-HTP: 5-hydroxytryptophan. A: Chromatogram of tyrosine to detect PAH activity. AAAH activity was determined in 3 ml reactions containing 82.6 µg phenylalanine as substrate and 45 µg purified protein. B: Chromatogram of 5-hydroxytryptophan to determine TRH activity. AAAH activity was measured in 3 ml reaction volume containing 100 ng of the substrate tryptophan and 75 µg purified protein. C: Quantity of the corresponding product produced per gram protease was plotted as the mean ± SD (n = 3).

To investigate the biochemical characteristics of *BmPAH*, we measured various enzymatic activities of the purified protein. These reactions were performed by addition of excess substrates to the purified protein along with the cofactors required for AAAH activity (see Materials and Methods). After reaction termination, corresponding end-products were analyzed with HPLC-FLD. The reaction mixture with phenylalanine and only 45 µg of the recombinant protein yielded ∼3 mg of tyrosine end-product/g protein (Figure 2A; Figure 2C). In contrast, the reaction containing L-tryptophan (tryptophan) as a substrate and 75 µg recombinant protein produced less 5-hydroxytryptophan end-product (Figure 2B; ∼70 µg 5-hydroxytryptophan/g recombinant protein; Figure 2C). In the reaction containing tyrosine as a substrate plus recombinant protein, dopa was not detected as an end-product (Figure 2C). These results reveal that recombinant BmPAH protein expressed *in vitro* had stronger PAH activity (converting phenylalanine to tyrosine), a weaker tryptophan hydroxylase (TRH or TPH, EC 1.14.16.4) activity, which converted some tryptophan to 5-hydroxytryptophan, and no tyrosine hydroxylase (TH) activity, which would have converted tyrosine to dopa. These are typical characteristics of PAH in invertebrates, indicating that the recombinant protein is indeed BmPAH. Note that in the reaction with tryptophan, more recombinant protein (75 µg instead of 45 µg) was used because 45 µg of the recombinant protein and tryptophan yielded an amount of 5-hydroxytryptophan that was below the threshold of computational detection.

### 2.2 Embryonic expression of *BmPAH*

**Figure 3:**
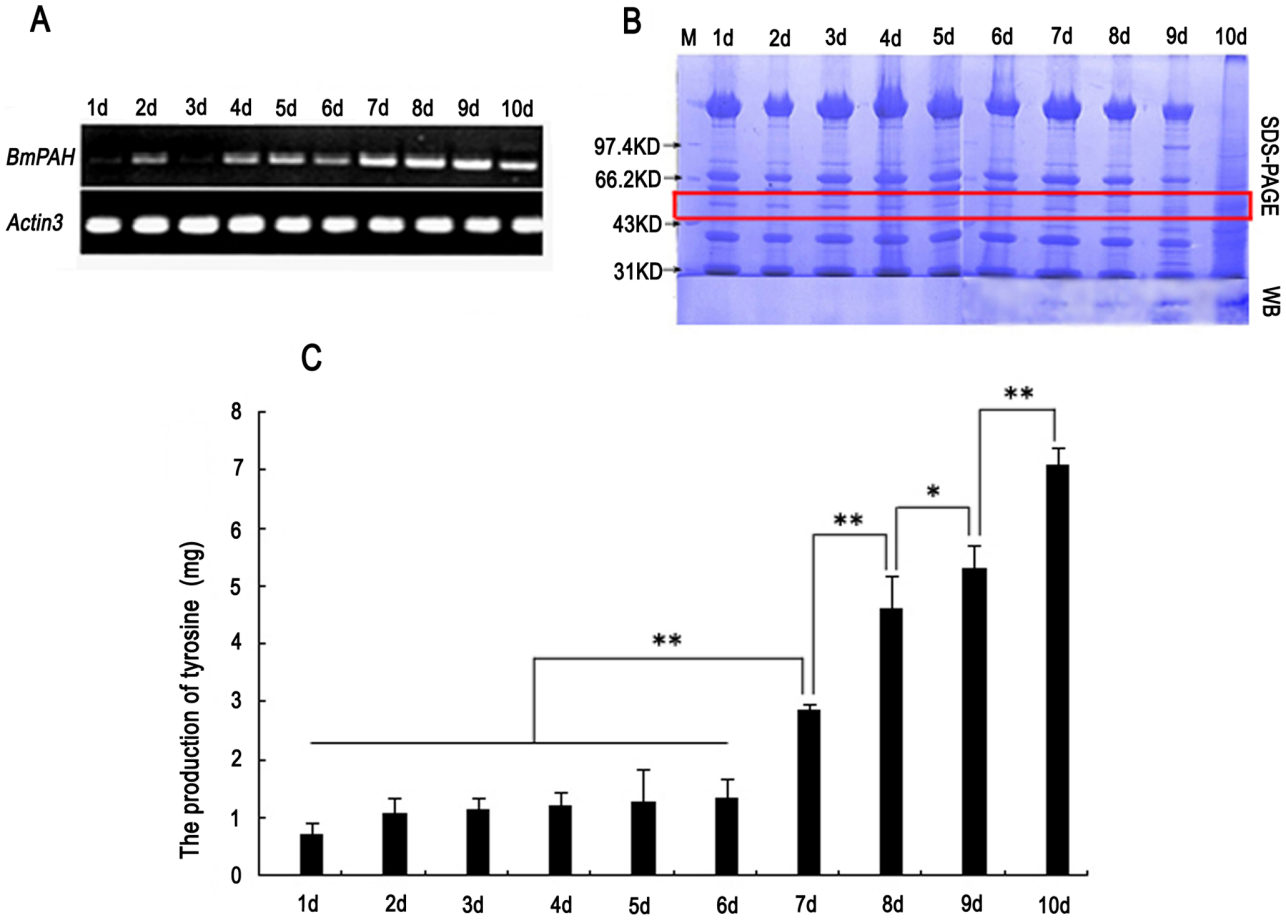
*BmPAH* transcripts, protein, and PAH activity in embryos. 1 d–10 d represent 1-day-old to 10-day-old embryos. A: Expression profile of BmPAH during embryonic development was determined by RT-PCR. *BmActin3* was used as the internal control. B: BmPAH protein was measured with 12% SDS-PAGE and Western blotting (WB). Each lane contained 100 µg total protein extracted from embryo samples. Red box indicates the BmPAH protein on SDS-PAGE. Antibodies to BmPAH protein was produced in rabbits. C: Production of tyrosine recorded during PAH activity analysis by HPLC-FLD. AAAH activity was analyzed in 3 ml reaction volumes containing 1,200 µg total protease extracted from embryos and 155 µg of the substrate, phenylalanine. Tyrosine produced per gram of total protease is plotted as the mean ± SD (n = 3). Each reaction was repeated three times. For statistical significance, data from 7 d were compared with data from 1 d–6 d; 8 d were compared to 7 d; 9 d were compared to 8 d; 10 d were compared to 9 d. * indicates significant differences at P<0.05; ** indicates significant differences at P<0.01.

Silkworm embryos synthesize large amounts of melanin to darken the head, epidermis, and setae 2–3 days before hatching. Semi-quantitative RT-PCR revealed less *BmPAH* during early embryonic stages, followed by a gradual increase from day 2 to day 6, and a significant up-regulation after day 6 (Figure 3A). Thus, *BmPAH* mRNA expression increases as embryos develop.

BmPAH protein was also detected from total protein during embryonic development, but BmPAH protein was not be detected with Western blot in 1–6 day-old embryos. Small amounts of protein were detected on day 7. Subsequently, in 8–10-day-old embryos, BmPAH protein increased gradually (Figure 3B). The change in BmPAH protein over time was consistent with *BmPAH* mRNA expression profiles and the embryonic coloring process.

### 2.3 Detection of PAH activity in embryos

To investigate PAH activity during embryonic stages, we measured tyrosine generated by HPLC-FLD in AAAH reactions, which contained total protein extracted from the embryos and an excess of the phenylalanine substrate. All control reactions with embryos of different ages were also performed under the same conditions without phenylalanine. Because total protein may contain endogenous tyrosine, the final tyrosine content in each reaction was calculated by subtracting the tyrosine content of the corresponding control. The smallest amount of tyrosine was produced by 1 day-old embryos, and slightly more tyrosine was produced in 2–6-day-old embryos, indicating a minimal change in PAH activity during this period. However, tyrosine increased significantly on day 7 and continued to increase gradually until day 10, suggesting higher PAH activity during late embryonic stages (Figure 3C). Such changes in PAH activity were consistent with our findings with semi-quantitative RT-PCR and Western blot during the embryonic stage, and suggested that *BmPAH* was involved in melanin synthesis for embryonic coloring.

### 2.4 Functional characterization of *BmPAH* in embryo coloring

**Figure 4:**
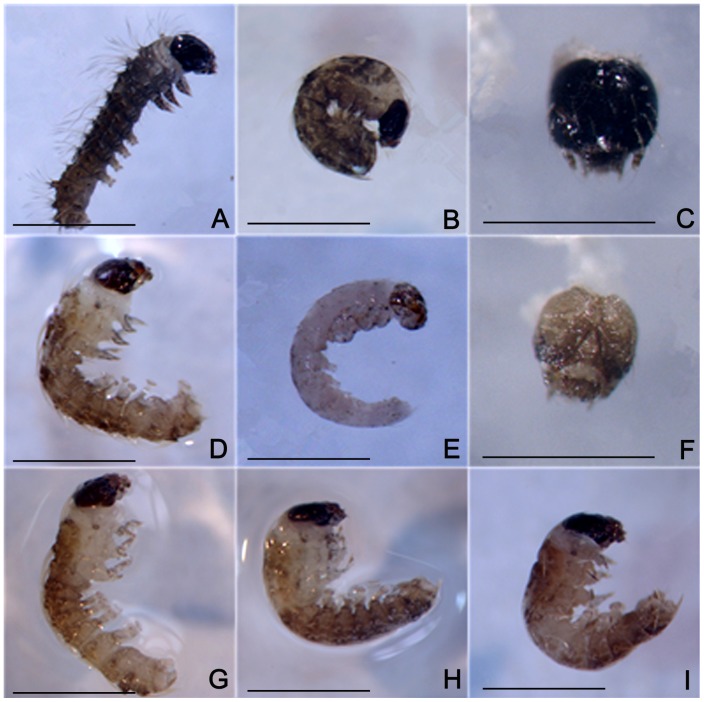
RNAi effects on coloration in GaoBai neonate larvae. Bar represents 1 mm. A–C: Phenotype of controls. Normally colored hatched neonatal larvae (A), unhatched neonatal larvae (B) and head (C). D–I: Phenotype of neonatal larvae with uneven coloring. D: Whole body with irregular and shallow color. E: Whole body without color except for the head with shallow coloring. F: Head colored shallow. G: Color in the posterior part of the body more shallow than the anterior part of the body. H: Color in the anterior part of the body more shallow than the posterior part of the body. I: Middle part of the body scarcely colored.

To determine whether a decrease in *BmPAH* mRNA caused unsuccessful embryo coloration, we injected eggs from the diapause-free GaoBai strain with *BmPAH* dsRNA. Approximately 3 nL of a solution containing dsRNA-1 or dsRNA-2, which corresponded to different regions in the *BmPAH* ORF, were injected in each GaoBai egg within three hours after the eggs were layed. Control eggs were injected with about 3 nL of RNAase-free water under the same conditions. Injected eggs were incubated at 25°C in a moist Petri dish until hatching. The first to hatch were the controls with a hatching percentage of 95.68%. The eggs injected with dsRNA hatched two days later with the hatching percentage of 47.27% and 53.34% in the dsRNA-1 and dsRNA-2 groups respectively, which were significantly lower than control. No significant differences in body color were observed between the neonatal larvae that hatched successfully in the injected group and the un-injected group. However, microscopic inspection and dissection of unhatched eggs revealed more unsuccessfully colored neonatal larvae in the dsRNA-1 (31.88%) and dsRNA-2 (24.22%) injected groups (Table 1). In these neonatal larvae, the forepart, posterior, or middle part of the body were scarcely colored and the head had modest coloring (Figure 4). This was not observed in the control. In addition, compared with the group injected with a lower concentration of dsRNA-2, the group injected with higher concentration of dsRNA-1 had a lower percentage of hatchability and a higher percentage of unsuccessfully colored neonate larvae (Table 1). This implied that the ratio of unsuccessfully colored neonate larvae could be dose-dependent. In these reactions, a less concentrated dsRNA-2 solution was used (3,108 ng/µl) compared to the dsRNA-1 solution (4,022 ng/µl), because eggs injected with a higher concentration dsRNA-2 (4,022 ng/µl) had more growth arrest which resulted in higher numbers of unhatched eggs or egg death. Therefore, the amounts of dsRNA-2 were reduced for the experiments.

**Table 1:** Detailed data for *BmPAH* RNAi of GaoBai eggs.

Type of dsRNA fragment injected	dsRNA concentration (ng/µl)	Eggs injected (N)	Unhatched eggs (N)	Unsuccessfully colored individuals (N)	Unsuccessfully colored individuals (%)
RNase-free water	0	278	12	0	0
dsRNA-1	4,022	696	367	177	31.88
dsRNA-2	3,108	633	289	70	24.22

**Figure 5:**
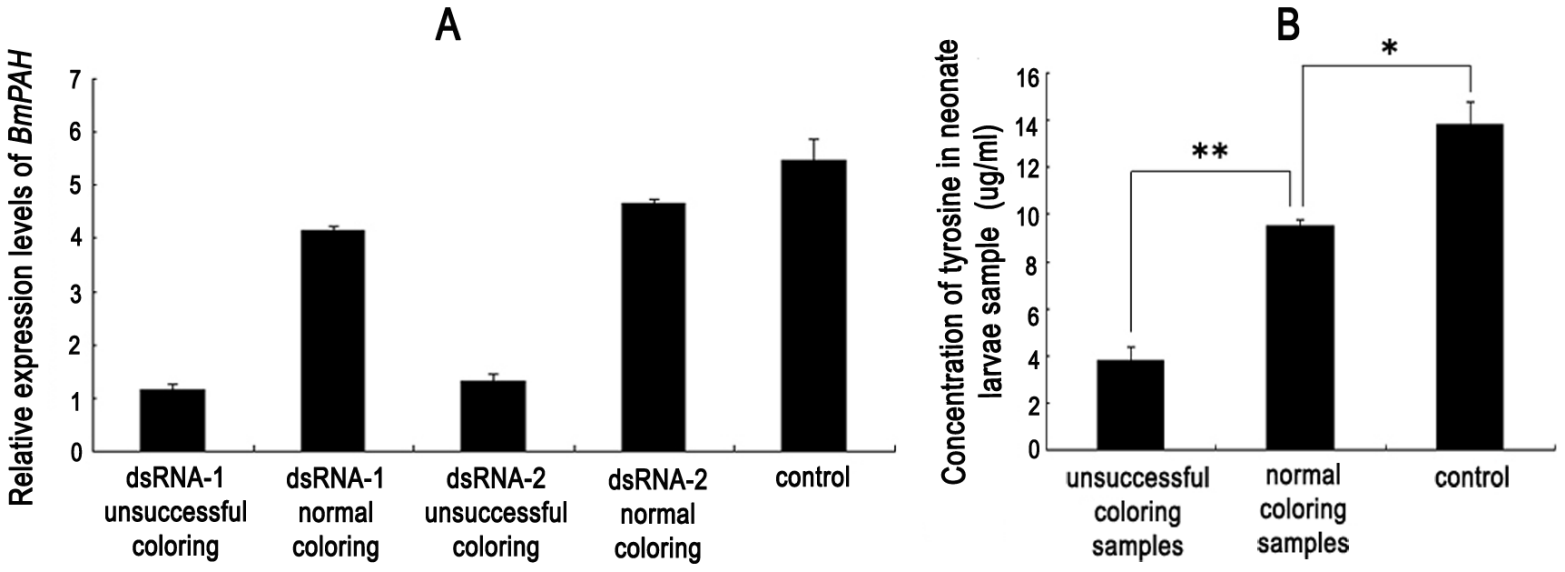
Effects of *BmPAH* RNAi on GaoBai neonatal larvae plotted as mean ± SD (n = 3). A: The relative expression level of *BmPAH* mRNA by quantitative RT-PCR. Eukaryotic translation initiation factor 4A (silkworm microarray probe ID: sw22934) was used as an internal control. dsRNA-1 and dsRNA-2 induced unsuccessful coloring or normal coloring in neonatal larvae after injection of eggs with dsRNA-1 and dsRNA-2. B: Concentration of tyrosine from neonate larvae. Unsuccessfully colored samples were from unsuccessfully colored neonatal larvae arising from eggs injected with dsRNA-1 and dsRNA-2, and normal coloring samples were from normally colored neonatal larvae arising from eggs injected with dsRNA-1 and dsRNA-2. * represents significant differences at P<0.05; ** represents significant differences at P<0.01.

Quantitative RT-PCR analysis showed a slight difference in *BmPAH* mRNA between the unsuccessfully colored neonatal larvae from the unhatched eggs of dsRNA-1 and dsRNA-2 injected groups as well as between the normally colored neonatal larvae from the eggs of dsRNA-1 and dsRNA-2 injected groups. When compared to controls, *BmPAH* mRNA in the normally colored neonatal larvae from dsRNA-1 and dsRNA-2 injected groups was only marginally lower. When eggs were injected with either dsRNA-1 or dsRNA-2, mRNA in unsuccessfully colored neonatal larvae was significantly lower (about 25%) of that measured in normally colored neonatal larvae and was ∼20% of that measured in control larvae (Figure 5A). These data show that unsuccessfully coloration in neonatal larvae was related to lower *BmPAH* expression.

Tyrosine, is a precursor for melanin synthesis and a product of PAH activity. Therefore, we measured tyrosine content separately in the unsuccessfully colored neonatal larvae and normally colored neonatal larvae arising from dsRNA-1 and dsRNA-2 injected egg groups. The unsuccessfully colored samples and the normally colored samples were further divided into three groups each. Because the number of individuals from different samples groups was different, the samples were quantified based upon the number of individuals. HPLC-FLD revealed significantly less tyrosine content in the unsuccessfully colored samples, compared to normally colored samples. Tyrosine content in unsuccessfully colored samples was 3.7 µg/ml, which was ∼30% of that measured in normally colored samples and ∼25% of that measured in control samples (Figure 5B). These results were consistent with our findings using quantitative RT-PCR and confirmed that the phenotype associated with unsuccessful coloration of neonatal larvae was directly related to low *BmPAH* mRNA. Collectively, with the abovementioned embryo analyses, our data indicate the participation of *BmPAH* in melanin synthesis for embryonic coloring.

### 2.5 *BmPAH* expression during molting

**Figure 6:**
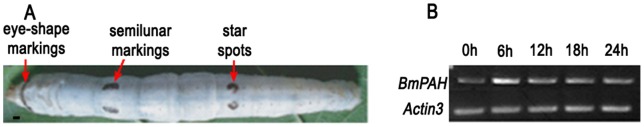
A: The dorsal view of DaZao 5^th^ instar larvae. B: The expression profile of *BmPAH* mRNA in the integument of 4^th^ instar larvae during molting from 0 to 24 h. *BmActin3* gene was used as an internal control.

During molting, markings that form on the new epidermis require abundant melanin, which is the main component of marking pigments in silkworm larvae. These markings are clearer and therefore easier to observe after the 4^th^ instar molting. Temporal expression patterns of *BmPAH* were observed in the larval integument during 4^th^ instar molting stage by semi-quantitative RT-PCR. *BmPAH* was expressed during the entire 4^th^ instar molting stage with relatively high expression at 6 h after the onset of 4^th^ molt (Figure 6B).

### 2.6 Effect of PAH inhibitors on larval markings

DaZao larvae have normal markings, which include pairs of dark brown eye-shaped markings, semilunar markings, and star spots distributed respectively in the 2^nd^, 5^th^ and 8^th^ segments on both sides of the dorsal midline (Figure 6A). To verify whether reduced PAH activity influenced the colors of these markings, we cultured larval integuments *in vitro* with or without a PAH inhibitor.

**Figure 7:**
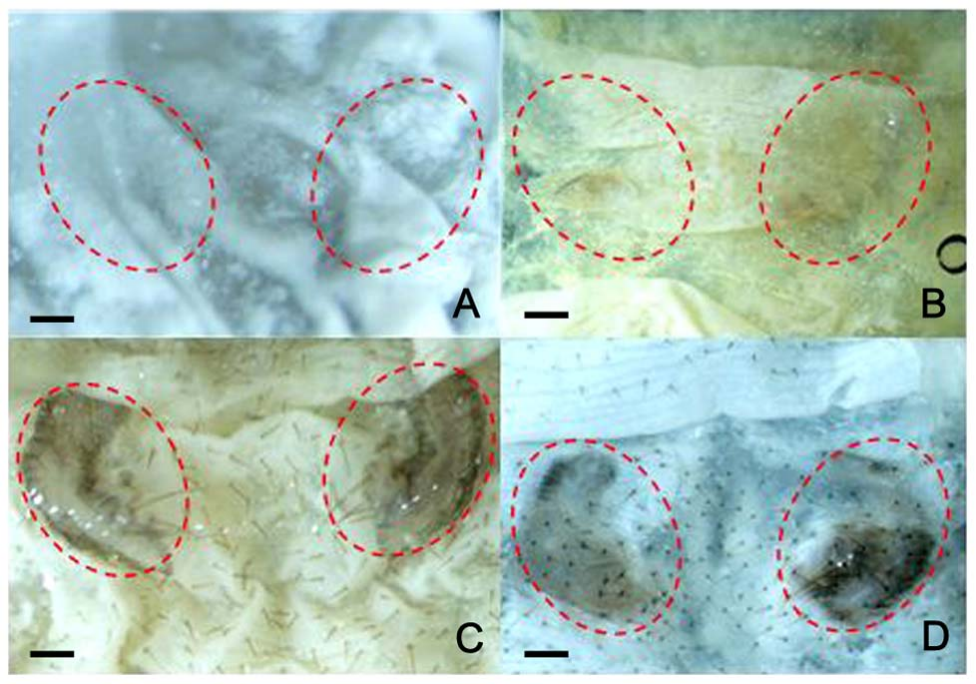
*In vitro* culture of integuments to determine the effectiveness of PAH inhibitors on the coloring of semilunar markings. Bar represents 1 mm. A: Integuments were just removed the old epidermis for culture. Positions of uncolored semilunar markings. B: Uncolored integuments at 24 h post-culture. The PAH inhibitor, esculetin, was added to the medium (10 mg/ml). C: Integuments with coloring in 24 h post-culture. Medium was added with tyrosine and esculetin (10 mg/ml). D: Control with normal coloring at 24 h post-culture. Medium lacked esculetin and tyrosine.

For this experiment, the integument was dissected from the 4^th^ instar larvae at 10–12 h after the onset of molting and the positions of the complete semilunar markings were noted, followed by removal of the old epidermis (Figure 7A). Unpigmented integuments after removal of the old epidermis were then subsequently cultured for 24 h at 26 °C in the culture medium. In controls containing only the culture medium, the markings were normally colored (Figure 7D), suggesting that the integument contained the precursor for melanin synthesis. When the PAH inhibitor, esculetin, was added to the medium, the markings had little or no color (Figure 7B), indicating that esculetin inhibited markings coloration most likely by interfering with conversion of the melanin precursor into melanin. In addition, when tyrosine was added in the presence of esculetin, marking coloration returned (Figure 7C) suggesting rescue of esculetin-inhibited pigmentation by tyrosine.

Therefore, esculetin possibly impeded melanin synthesis by preventing the conversion of the melanin precursor into tyrosine. These data show that PAH activity is essential for cuticular pigmentation and that phenylalanine is an important precursor of melanin synthesis in the silkworm integument.

**Figure 8:**
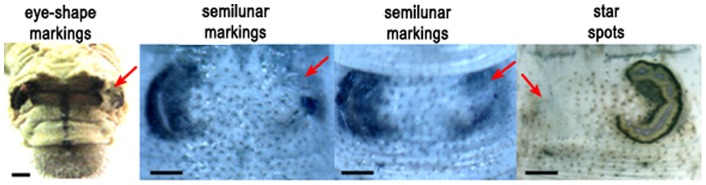
The phenotype of discolored markings (eye-shaped markings, semilunar markings, star spots). Red arrow indicates discolored markings after esculetin injection. Bar represents 1 mm.

Next, we injected 30 µg/µl esculetin into one side of the eye-shaped markings, semilunar markings, and star spots of 4^th^ instar larvae during the enlargement period of the thorax prior to molting. Compared to the un-injected side, markings on the injected side partially or completely disappeared after the 4^th^ instar larval molt (Figure 8). To ascertain the dose-response effect on marking discoloration, we injected 150 µg (5 µl), 300 µg (10 µl) or 450 µg (15 µl) esculetin into semilunar markings. In the controls, 15 µl solvent was injected into semilunar markings under identical conditions. No difference was observed between the control and the group that received 150 µg esculetin (Table 2). However, esculetin at 300 µg decreased coloration in 20% of the samples, and 70% of the samples had reduced coloration with 450 µg esculetin (Table 2).

**Table 2:** Effect of PAH inhibitors on semilunar markings.

Esculetin (µg)	Larvae injected (N)	Vital larvae (N)	Discolored individuals (N)
0	10	10	0
150	13	13	0
300	10	10	2
450	14	10	7

*In vitro* culture and injection of the integument revealed that PAH activity was essential for melanin synthesis. Together with the mRNA expression profile in the larval integument during the 4^th^ instar molting stage, these data show that *BmPAH* is necessary for coloring of larval markings. Based on the biological characteristics of silkworm coloring, our results suggested that *BmPAH* is needed for melanin synthesis and that phenylalanine is an important precursor of melanin synthesis in the silkworm.

## Discussion

In this study, we isolated *BmPAH* from *B. mori* and showed that the resultant BmPAH protein expressed in a heterologous system is indeed a PAH homolog because of its primary ability to convert phenylalanine to tyrosine. BmPAH also had weaker TRH activity and no TH activity, which are typical characteristics of invertebrate PAH. During embryogenesis, both *BmPAH* and BmPAH increased as embryos developed. Furthermore, silencing *BmPAH* indicated a role for this gene in melanin synthesis, a crucial event in silkworm body coloration.

PAH is present in all metazoans, and its amino acid sequence is highly conserved among species separated by a large evolutionary distance [32], suggesting a need for PAH in living organisms. In lower animals such as the sponge, *Geodia cydonium*, PAH was reported to be involved in melanin formation used in the immune response [33] and in *Caenorhabditis elegans* PAH plays a role in epidermal pigmentation [34]. In insects, *PAH* was proposed to be involved in melanin synthesis towards immune response (Nappi *et al*. [6], Zhao *et al.* [7] and Huaping T [35]). Further, the immune response of *Epirrita autumnata* against abiotic foreign objects showed that phenylalanine was the substrate for melanin synthesis [36]. In addition, when *Exorista sorbillans* Wiedemann parasitized silkworm larvae, hemolymph melanization was induced as evidenced by darker hemolymph [37]. Our data that confirm a role for PAH in melanin synthesis as indirectly demonstrated in the immune response of *B. mori*.

In addition, previous gene knockdown experiments in mosquitoes *Aedes aegypti* and *Armigeres subalbatu* suggested a requirement for PAH for a fully functional melanotic encapsulation response to microfilariae [8]. Interestingly, knockdown of PAH had no effect on Sephadex bead melanization in *Anopheles gambiae* [38]. Using RNAi-mediated *BmPAH* gene silencing we showed a reduction in tyrosine in neonatal larvae that caused unsuccessful coloring, suggesting the role of *BmPAH* in body coloring. Moreover, decreasing PAH activity with a PAH inhibitor caused loss of coloration of larval markings, clearly suggesting the participation of BmPAH in melanin synthesis and subsequent coloration. This is unique with respect to *Papilio xuthus* for which expression of *PAH* mRNA did not coincide with melanin synthesis with respect to eye-shaped markings in the 5th instar [39]. In *D. melanogaster*, *Henna* encoding PAH was involved in the synthesis of pteridines and the formation of the eye pigment pterin [40]–[41], although it was not clear which of the two PAH subtypes was linked to this function. The two subtypes of the PAH homolog in *D. melanogaster* had significantly different expression patterns [42] suggesting different functions. However, in the silkworm genome, we have thus far identified only a single copy of BmPAH and no subtypes, a finding similar to that in humans and some invertebrates [27], [33], [39], [43]–[44].

**Figure 9:**
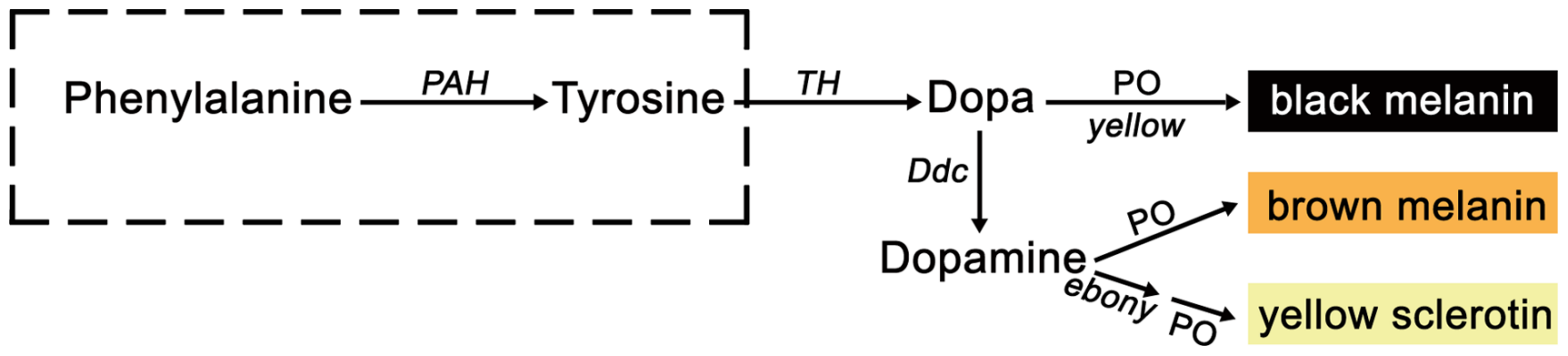
Model of pigment metabolism in silkworm melanogenesis (11, 45–47). Precursors, intermediates, and pigments are shown at ends of arrows, and enzyme or genes are shown at the side of arrows. The new step added is shown within wire frame. *PAH*: phenylalanine hydroxylase gene; *TH*: tyrosine hydroxylase gene; *Ddc*: dopa decarboxylase gene; *ebony*: N-β-alanyldopamine synthetase gene; *yellow*: royal jelly protein gene; PO: phenoloxidase.

Initial studies into the pigment anabolism pathway in *D. melanogaster* promoted a number of studies to investigate melanin synthesis in other insects which are similar to *D. melanogaster* [4], [11]–[13], [17]. Genetic information from genomes of sequenced silkworm together with functional genomics [45]–[47] has contributed to the current view of melanin biosynthesis, which is initiated by the conversion of tyrosine into dopa by the action of TH. This reaction was also confirmed as an essential step in melanin biosynthesis [48], which was also thought to be the initial step in melanin anabolism pathways in silkworm. Our data show that conversion of the essential amino acid phenylalanine to tyrosine by PAH is likely to be the primary step in melanogenesis, and therefore phenylalanine – and not tyrosine – is the initial precursor for melanin biosynthesis in *B. mori*. Our study confirms a previously unknown step in the melanin anabolism pathway in the silkworm. Specifically, PAH catalyzes the hydroxylation of phenylalanine to tyrosine that is the first step in the melanin anabolism pathway (Figure 9), which is similar to mosquitoes.

## Materials and Methods

### 4.1 Ethics Statement

All experiments involving animals were approved by the Committee of Laboratory Animal Welfare and Ethics, Southeast University (Permit Number: 2010–026). All procedures followed the regulations issued by the review committee of laboratory animal welfare and ethics and the protocol for the review on laboratory animal welfare and ethics, Southeast University.

### 4.2 Silkworm Strains

The diapause-free GaoBai strain was used in all experiments involving egg injections and the DaZao strain was used in the remainder of the experiments. Neonatal larvae of both strains were black.

### 4.3 mRNA isolation and cDNA synthesis

Total RNA was purified using TRIzol reagent (Invitrogen) according to the manufacturer's instructions, and cDNA was synthesized using oligo (dT) primers and Moloney murine leukemia virus (M-MLV) reverse transcriptase (Promega).

### 4.4 Cloning

cDNA from the heads of 5^th^ instar DaZao larvae were used as PCR templates to amplify *BmPAH* using primers F5′-ACACGCCTCTTCCAGCAACA-3′ and R5′-GTCCCACCTACAACAAGAAGCTAAT-3′. PCR reaction conditions included 94°C for 3 min, 30 cycles of 94°C for 30 s, 55.5°C for 1 min and 72°C for 1.5 min and a final extension at 72°C for 10 min. PCR products were isolated and cloned into the pMD18-Tsimpe vector (Takara) and sequenced.

### 4.5 Recombinant protein expression and purification

Based on the instructions of the Bac-to-Bac Baculovirus Expression System (Invitrogen), the complete coding sequence of *BmPAH* was recombined into the pFastBac ™ HT-A vector and Bacmid baculovirus plasmid (Figure S1). The recombinant Bacmid plasmid was then transfected into Sf9 cells (Invitrogen) (Figure S2) for protein expression (Figure S3). The recombinant protein containing 6× His tag was purified by affinity chromatography using an Ni^2+^column. The purified protein was then quantified using the Bradford method.

### 4.6 Mass chromatographic analysis and antibody preparation

Purified protein was analyzed by MALDI-TOF-MS as described by Hou *et al.* [49].

#### Local protein database construction and protein retrieval

Data were primarily downloaded from the silkworm genome database (http:silkworm.Swu.edu.Cn/silkdb) and data from the NCBI protein database was used as a supplement. Peptide mass fingerprinting was analyzed by GPMAW software [50]. The parameters used were as follows: precision  = 0.10%; min. prec  = 0.50 Da; min. hits  = 2; max. overlap  = 2. Identification criteria were based on the number and coverage of matched peptides: a minimum of 5 peptides were required to match and the coverage of the matched peptides was ≥25%.

The purified protein was used to immunize healthy male adult rabbits. Antibodies from these rabbits were extracted and purified by the saturated ammonium sulfate method using the MabTrap Kit (GE Healthcare) according to the manufacturer's specifications. The antibody titer was then measured by ELISA (Sigma) and was 1∶6,000.

### 4.7 Activity assay for AAAH

Enzyme activity was measured as described by Wang *et al.* [51] using phenylalanine, tryptophan, and tyrosine as substrates (Sigma). Reactions were terminated with trichloroacetic acid after 40 min at 30°C, and the product was detected after filtration by high performance liquid chromatography with fluorescence detection (HPLC-FLD) as described by Sa and co-workers [52]. In these assays, tyrosine, 5-hydroxytryptophan, and dopa (Sigma) were used as standards, and the standard chromatograms are depicted in the supporting information (Figure S4).

### 4.8 Semi-quantitative RT-PCR for expression analysis

Expression analysis by RT-PCR was performed using primers F5′-AGTGTTCCACAGCACCCAGTA-3′ and R5′-TTGTCCATAGCGTTTAGCAG-3′ that amplified 567 bp. Conditions of PCR consisted of 94°C for 3 min, 25 cycles at 94°C for 30 s, 51°C for 40 s and 72°C for 1 min and a final extension at 72°C for 10 min. Templates for the reaction were cDNA from embryos or larval integuments from the 4^th^ molting stage. *BmActin3* was used as an internal control.

### 4.9 Quantitative RT-PCR

Quantitative RT-PCR was performed on an ABI Prism 7000 sequence detection system (Applied Biosystems) using SYBR green. The primers used for *BmPAH* were: F5′-ATCCCATCACAGAATACCAG-3′ and R5′-TGTCCATAGCGTTTAGCAG-3′. Internal control primers for the eukaryotic translation initiation factor 4A (silkworm microarray probe ID: sw22934) were: F5′-TTCGTACTGGCTCTTCTCGT-3′ and R5′-CAAAGTTGATAGCAATTCCCT-3′.

### 4.10 RNAi

Primers for the synthesis of dsRNA-1 were based on 1–600 bp of the ORF and amplified a 537-bp product. Primers for the synthesis of dsRNA-2 were based on sequences from 800–1,350 bp of the ORF and the length of the fragment amplified was 553 bp (Table S1). Concentration and purity of the synthetic DNA fragments used as template and dsRNA fragments were determined using a BECKMAN DU-600 spectrophotometer. dsRNA synthesis and purification were carried out as described in the MEGA script RNAi Kit. Integrity of dsRNA was confirmed by nondenaturing agarose gel electrophoresis.

Silkworm eggs of the GaoBai strain were used for dsRNA microinjection following the method described by Quan *et al*. [53]. Neonatal larval color was observed and digitally recorded using a SMZ850 microscope (Touptek).

To detect tyrosine by HPLC-FLD, neonatal larvae were ground and the protein was precipitated with 5% HClO_4_ (Sigma). Samples were then centrifuged at 15,000 rpm/min at 4°C to remove protein. Based on the number of neonatal larvae, samples were quantified such that 10 µl of 1% HClO4 was present per neonatal larvae for tyrosine detection.

### 4.11 In vitro culture and injection of integuments

After removal of fat bodies, muscles, and trachea, the new epidermis was washed thoroughly in 0.01 M PBS (pH 7.2), and then cultured in Grace's medium containing 12% fetal bovine serum. The medium did not exceed more than one half of the 2 ml culture flask volume.

For injections, esculetin was dissolved in 0.015 M NaHCO_3_ to a final concentration of 30 µg/µl. Larval phenotypes were observed after the 4^th^ molt and pictures were obtained under a SMZ850 microscope (Touptek).

## Supporting Information

Figure S1 **The recombinant Bacmid baculovirus plasmid was detected by PCR using M13 primers.** Red arrow indicates the target sequence. The length was about 2430 bp between left and right arm of Tn7 transposon, and 273 bp between primers. If transposition was successful, the expected fragment was approximately 4000 bp in addition to the target fragment; when no exogenous gene was inserted into Bacmid plasmid, the fragment was about 300 bp using M13 primers by PCR.
(TIF)

Figure S2 **Sf9 cells infected with the recombinant Bacmid baculovirus (72h post- infection).** Red arrow indicates floating cells, collapsing cells, etc.
(TIF)

Figure S3 **Western blot analysis of the soluble fraction of Sf9 cell lysate infected with** ***BmPAH*** **recombinant Baculovirus (72 h post infection).** Lane 1 – control, which is the soluble fraction from the cell lysate of Sf9 cells infected with control Baculovirus. Lane 2 – soluble fraction from the cell lysate of Sf9 cells infected with the recombinant Baculovirus. Primary antibody: Mouse anti-His antibody, Secondary antibody: Goat anti-mouse HRP. (Molecular weight of the predicted protein encoded by the BmPAH gene was about 52 KDa. Addition of the His tag increased the molecular weight to about 55 KDa).
(TIF)

Figure S4 **Standard chromatogram of L-tyrosine, L-Dopa and 5-hydroxytryptophan (Sigma). Tyr – L-tyrosine; 5-HTP – 5-hydroxytryptophan; Dopa – L-dioxyphenylalanine.**
(TIF)

Table S1 **Primers for the synthesis of the dsRNA.**
(DOC)
