# Supplementary figures and images for: BmPAH Catalyzes the Initial Melanin Biosynthetic Step in Bombyx mori
Source: PLoS One. 2013 Aug 26;8(8):e71984. doi: 10.1371/journal.pone.0071984 (PMC3753331; doi:10.1371/journal.pone.0071984)

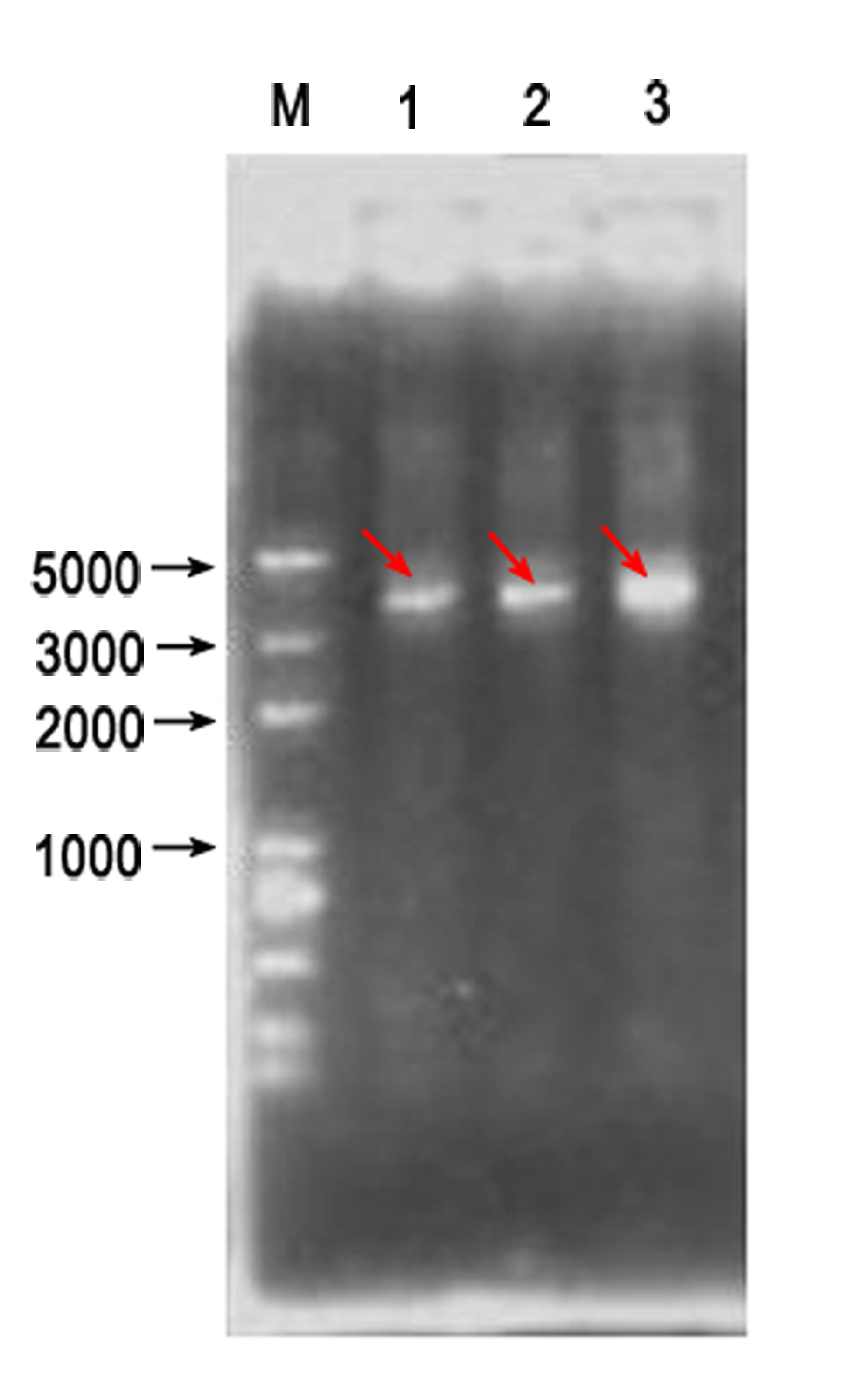

Supplement: Figure S1 — The recombinant Bacmid baculovirus plasmid was detected by PCR using M13 primers. Red arrow indicates the target sequence. The length was about 2430 bp between left and right arm of Tn7 transposon, and 273 bp between primers. If transposition was successful, the expected fragment was approximately 4000 bp in addition to the target fragment; when no exogenous gene was inserted into Bacmid plasmid, the fragment was about 300 bp using M13 primers by PCR. (TIF) [file pone.0071984.s001.tif]

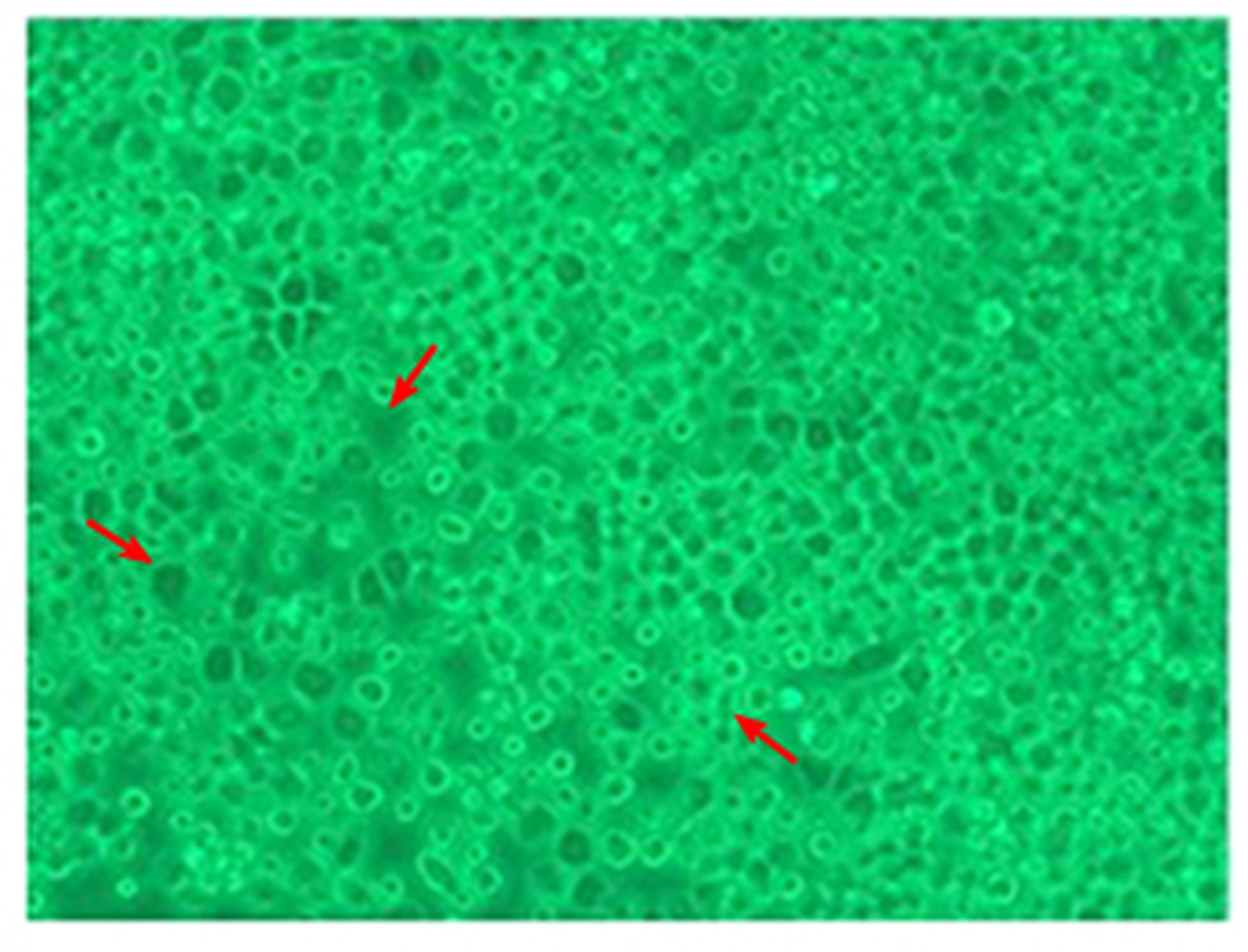

Supplement: Figure S2 — Sf9 cells infected with the recombinant Bacmid baculovirus (72h post- infection). Red arrow indicates floating cells, collapsing cells, etc. (TIF) [file pone.0071984.s002.tif]

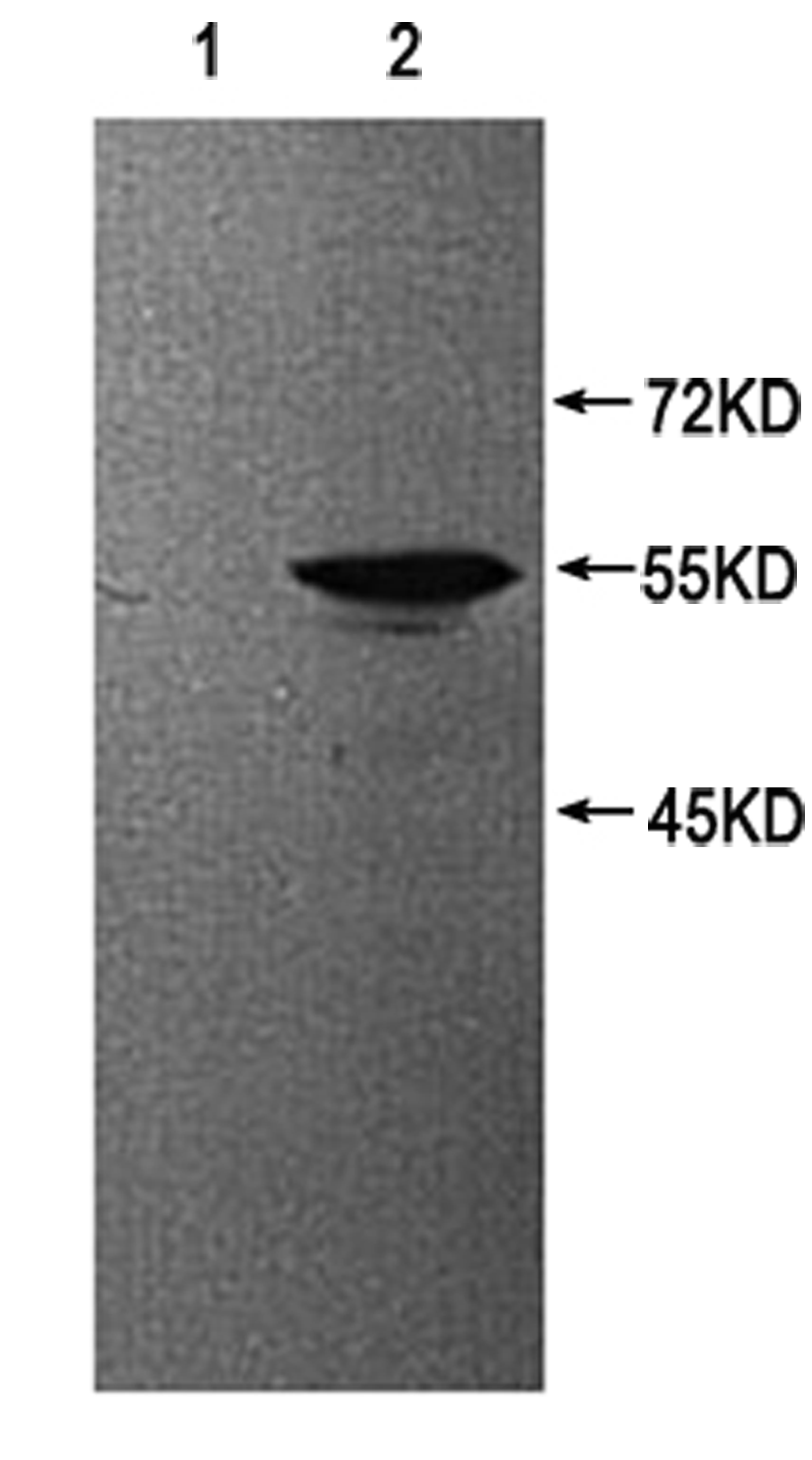

Supplement: Figure S3 — Western blot analysis of the soluble fraction of Sf9 cell lysate infected with BmPAH recombinant Baculovirus (72 h post infection). Lane 1 – control, which is the soluble fraction from the cell lysate of Sf9 cells infected with control Baculovirus. Lane 2 – soluble fraction from the cell lysate of Sf9 cells infected with the recombinant Baculovirus. Primary antibody: Mouse anti-His antibody, Secondary antibody: Goat anti-mouse HRP. (Molecular weight of the predicted protein encoded by the BmPAH gene was about 52 KDa. Addition of the His tag increased the molecular weight to about 55 KDa). (TIF) [file pone.0071984.s003.tif]

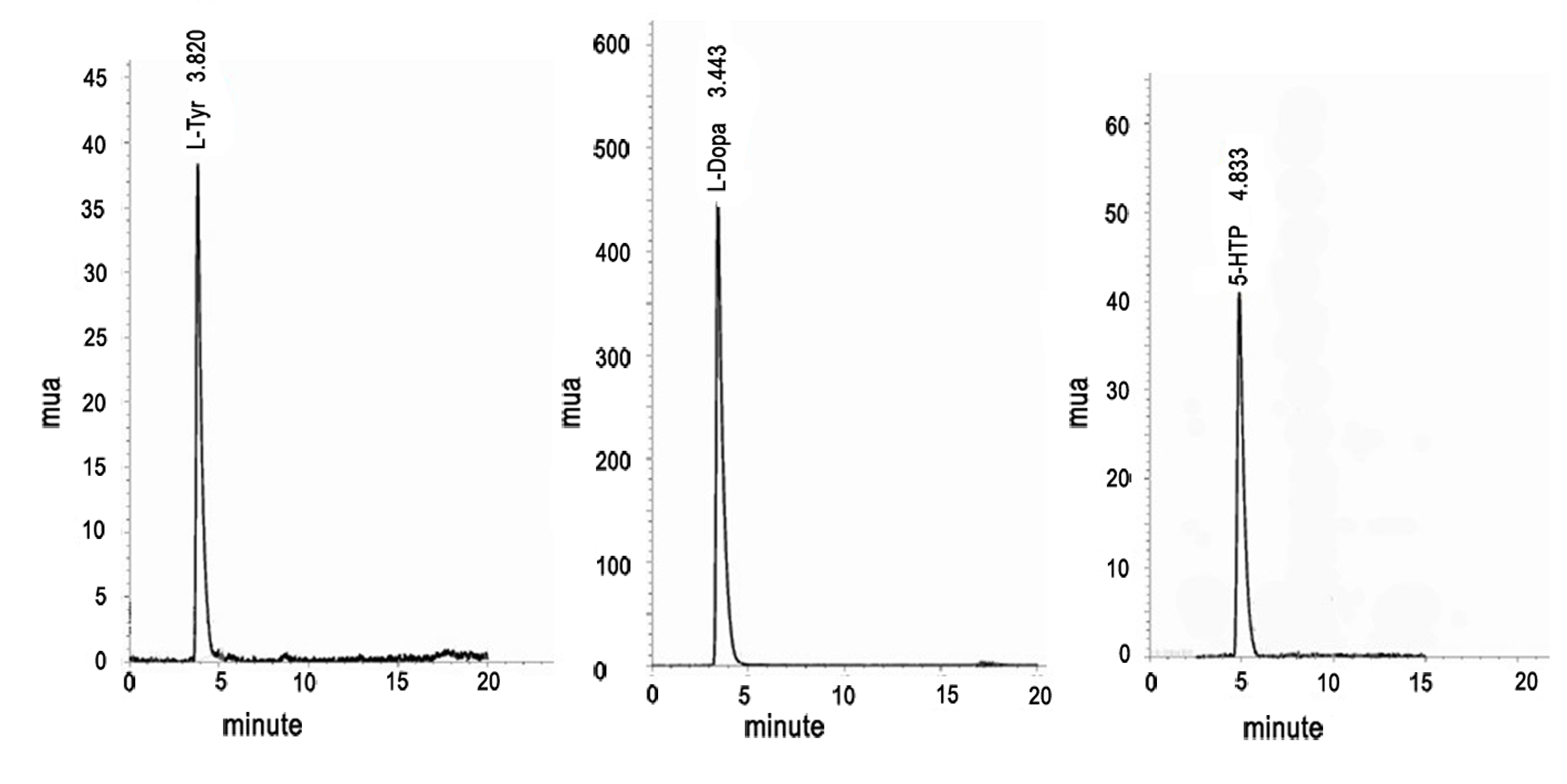

Supplement: Figure S4 — Standard chromatogram of L-tyrosine, L-Dopa and 5-hydroxytryptophan (Sigma). Tyr – L-tyrosine; 5-HTP – 5-hydroxytryptophan; Dopa – L-dioxyphenylalanine. (TIF) [file pone.0071984.s004.tif]
