# Supplementary material for: BmPAH Catalyzes the Initial Melanin Biosynthetic Step in Bombyx mori
Source: PLoS One. 2013 Aug 26;8(8):e71984. doi: 10.1371/journal.pone.0071984 (PMC3753331; doi:10.1371/journal.pone.0071984)
Supplement: Table S1 — Primers for the synthesis of the dsRNA. (DOC) [file pone.0071984.s005.doc]

**Table S1: primers for the synthesis of the dsRNA**

| Name | Primers | Sequences(5'→ 3') |
| --- | --- | --- |
| dsRNA-1 | F | 5‘ GTAATACGACTCACTATAGGGAGAGAAGCAAATTGAACAACCCAC 3 ‘ |
|  | R | 5‘ GTAATACGACTCACTATAGGGAGATACTCCACATAAGGCAGAGG 3’ |
| dsRNA-2 | F | 5‘ GTAATACGACTCACTATAGGGAGATGTTCCACAGCACCCAGTAT 3‘ |
|  | R | 5‘ GTAATACGACTCACTATAGGGAGATTTAGCAGCAGCTCCATTTC 3‘ |
